# Supplementary material for: Construction of a Triple-Gene Deletion Mutant of Orf Virus and Evaluation of Its Safety, Immunogenicity and Protective Efficacy
Source: Vaccines (Basel). 2023 Apr 28;11(5):909. doi: 10.3390/vaccines11050909 (PMC10224447; doi:10.3390/vaccines11050909)
Supplement: Supplementary file 1 [file vaccines-11-00909-s001.zip › vaccines-2288001-supplementary.pdf]

Supplementary materials

Table S1. Primers used to generate and identify the recombinant viruses.

Primers	Sequence (5'–3')	Descriptions
121-hm1 F	CGACATCGCACACAAATGAAGGACA	Used to amplify the left arm of rGS14ΔCBPΔGIFΔ121-EGFP
121-hm1 R	ACGTAGAAGACCAGGAAACAG GGTTGTGTGGGCCACAGAGTTGAGT	
121-hm2 F	ACTCATCAATGTATCTTAAGGCGTGAAAGTGCGTTTTTCTGTAAT GTGA	Used to amplify the right arm of rGS14ΔCBPΔGIFΔ121-EGFP
121-hm2 R	CGTAGCAGAACAGCGACAGC	
121-EGFP F	ACTCAACTCTGTGGCCCACACAACC CTGTTTCCTGGTCTTCTACGT	Used to amplify the EGFP marker gene
121-EGFP R	TCACATTACAGAAAAACGCACTTTCACGCCTTAAGATACATTGAT GAGT	
121-hm1-hm2 F	ACTCTGTGGCCCACACAACCGAAAGTGCGTTTTTCTGTAAT	Used to amplify the right arm of rGS14ΔCBPΔGIFΔ121
121-hm2-hm1 R	TTACAGAAAAACGCACTTTCGGTTGTGTGGGCCACAGA	Used to amplify the left arm of rGS14ΔCBPΔGIFΔ121
121 F	TAGGTGCGTTCAGAGGCG	Used to identify the 121 gene in ORFV
121 R	CGTAGCAGAACAGCGACAGC	

Table S2. Clinical scoring sheet.

	absence (0')	mild or small number (1')	moderate or medium number (2')	severe or high number (3')
hyperemia				
vesicles and/or pustules				
scabs				
exudation and/or bleeding				

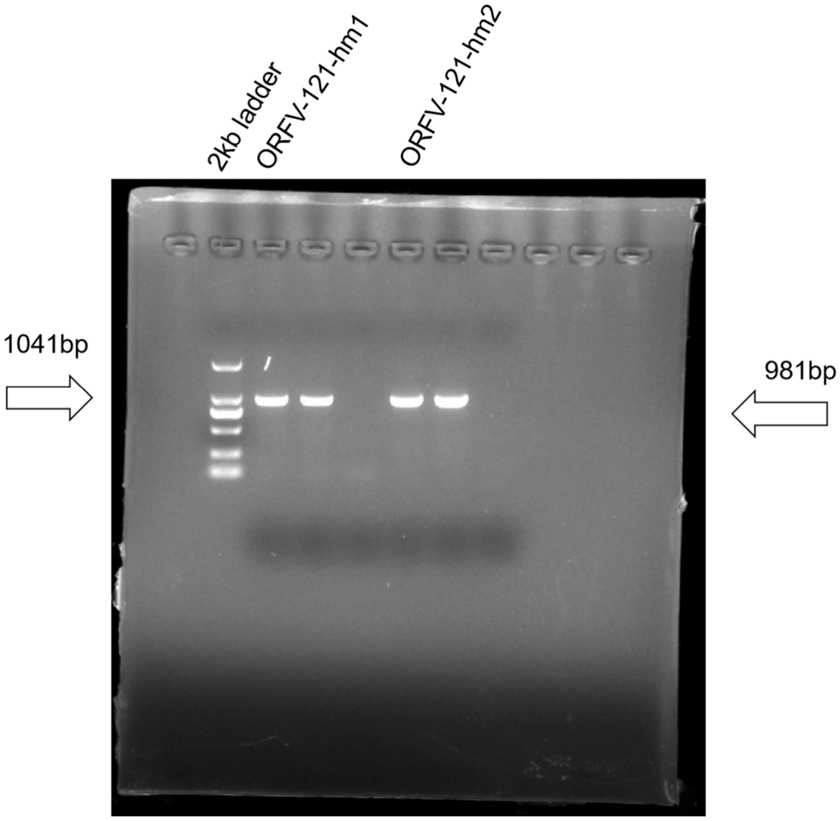

A

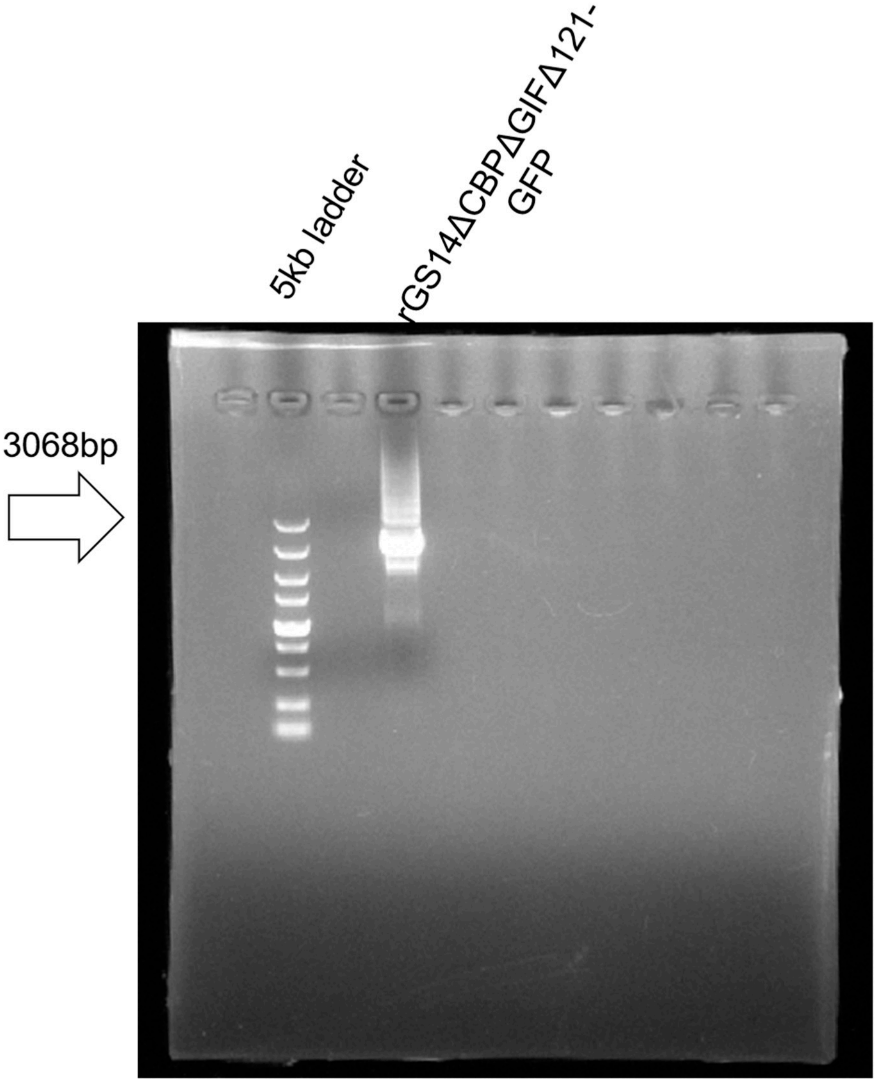
5kb ladder
rGS14ΔCBPΔGIFΔ121-
GFP
3068bp

B

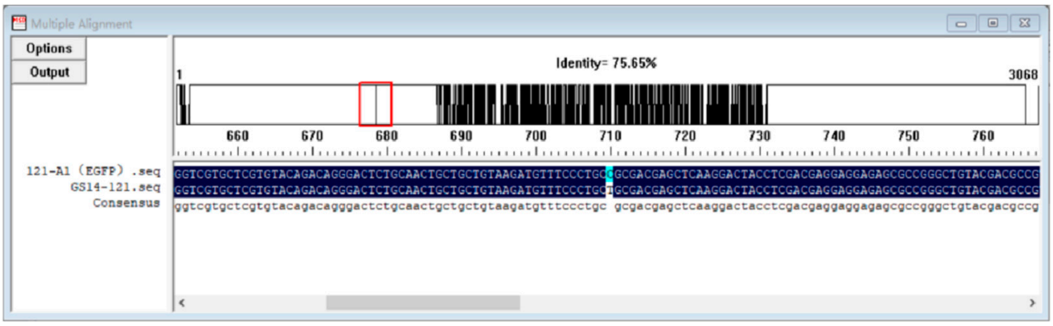
Multiple Alignment
Options
Output
Identity= 75.65%
1
3068
660
670
680
690
700
710
720
730
740
750
760
121-A1 (EGFP) .seq
GS14-121.seq
Consensus
ggtcgtgctcgtgtacagacagggactctgcaactgctgctgtaagatgtttccctgc gcgacgagctcaaggactacctcgacgaggaggagagcgccgggctgtacgacgccg

C

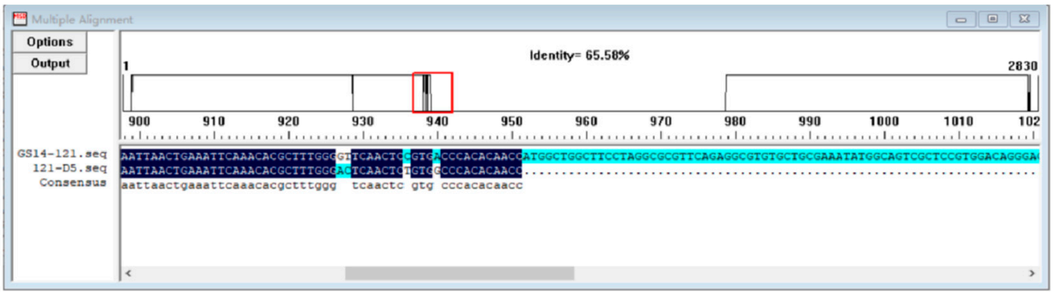

D

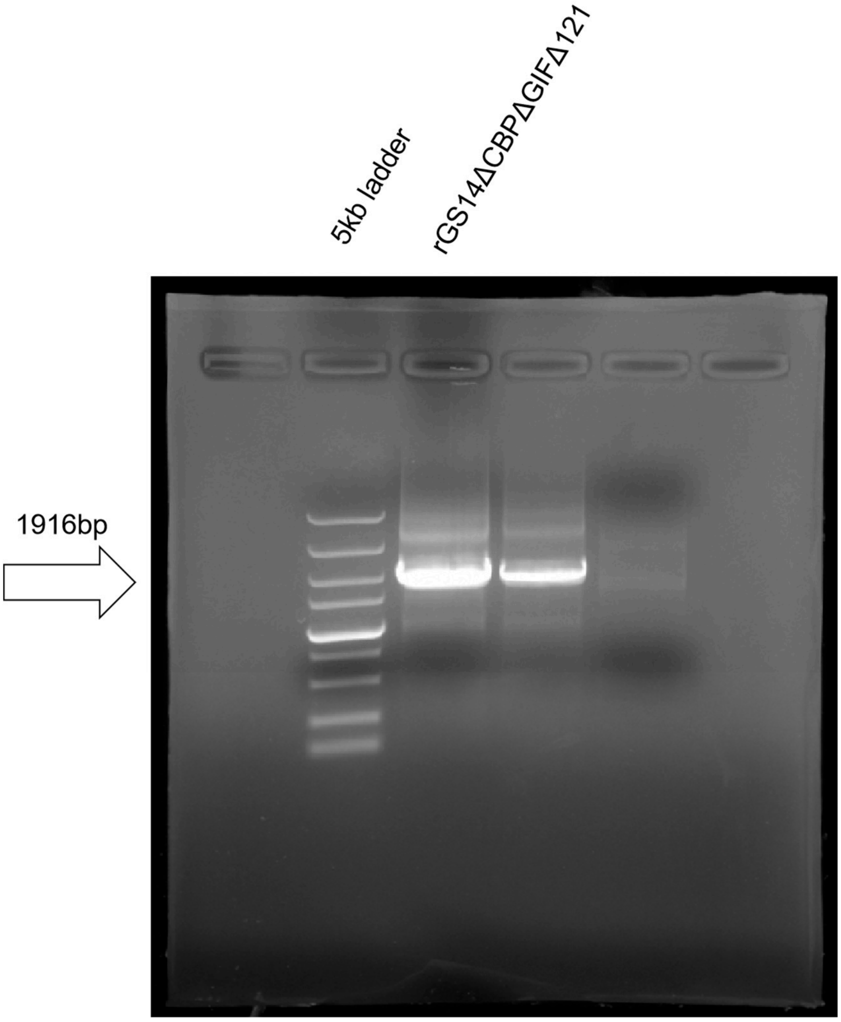

E

Figure S1. Partial identification results of recombinant virus rGS14ΔCBPΔGIFΔ121 construct. **A**: Bands showed the rGS14ΔCBPΔGIF containing 121 left homology arm and right homology arm.; **B**: The band showed the generation of rGS14ΔCBPΔGIFΔ121-GFP. **C**: Sequencing result of rGS14ΔCBPΔGIFΔ121-GFP compared with 121 gene of GS14. **D**: Sequencing result of rGS14ΔCBPΔGIFΔ121 compared with GS14-121. **E**: The band showed the construction of rGS14ΔCBPΔGIFΔ121
